# Three-candidate election strategy

**DOI:** 10.1098/rsos.230584

**Published:** 2023-09-27

**Authors:** Dorje C. Brody, Tomooki Yuasa

**Affiliations:** ^1^ Faculty of Natural Sciences, Department of Mathematics, Imperial College London, Exhibition Road, London SW7 2AZ, UK; ^1^ School of Mathematics and Physics, University of Surrey, Guildford GU2 7XH, UK; ^3^ 3Faculty of Economics and Business Administration, Graduate School of Management, Tokyo Metropolitan University, Tokyo 100-0005, Japan

**Keywords:** signal processing, electoral competition, stochastic filtering, measure change

## Abstract

The probability of a given candidate winning a future election is worked out in closed form as a function of (i) the current support rates for each candidate, (ii) the relative positioning of the candidates within the political spectrum, (iii) the time left to the election, and (iv) the rate at which noisy information is revealed to the electorate from now to the election day, when there are three or more candidates. It is shown, in particular, that the optimal strategy for controlling information can be intricate and non-trivial, in contrast to a two-candidate race. A surprising finding is that for a candidate taking the centre ground in an electoral competition among a polarized electorate, certain strategies are fatal in that the resulting winning probability for that candidate vanishes identically.

## 1. Introduction

This paper is concerned with informational strategies behind an election involving three or more candidates. Suppose that there is an election coming up next year, and that the three candidates have the support rates of, say, 38%, 26% and 36%, according to the current opinion poll statistics. Do these numbers represent the current likelihoods of the candidates winning the future election? If not, what are they? Can we derive a formula for a given candidate winning the election in the future? Because voter preferences change over time in accordance with information revealed to them, such a formula ought to be dependent on how information is managed between today and the future election day. In this paper, an exact formula will be worked out for the probability of winning a future election that depends explicitly on the model for the flow of information.

With such a formula at hand, we are able to ask a range of strategic questions and find quantitative answers. For example, for the candidate lagging behind with only 26% support today, in which way should they reveal policy information so as to maximize the realized probability of winning the future election? How would that differ from the strategy for other candidates? Are there advantages in the positioning of the candidate’s political party within the political spectrum, for example, leaning further to the right or to the left? Our purpose here is to provide a systematic framework to answer questions of this kind, building on the previous work on two-candidate election models [1–3]. In particular, we shall reveal some surprising features that emerge from having more than two candidates in an electoral competition.

It is worth remarking that there is a long history of building mathematical models to analyse various aspects of electoral competitions (e.g. [4–13]). The models hitherto considered in the literature tend to be either deterministic, or else probabilistic but static. While the importance of the role played by information in electoral competitions is widely acknowledged, the models that have been proposed in the literature omit the impact of noise, such as rumours, speculations, disinformation and so on. By contrast, our formulation, building on a successful application of the noisy information-based approach to modelling the dynamics of financial assets [14], takes on board the revelation of noisy information, which in turn can be used to deduce the statistics of the outcomes of future elections. In this way, a sensitivity analysis can be pursued so as to determine the impact of different informational strategies on the future statistics.

With these preliminaries, the present paper will be organized as follows. We begin with a brief introduction to the information-based approach to election modelling introduced in [1]. We then proceed by reviewing some of the key findings of [2] in which the probability of winning a future election is worked out in the case of a two-candidate electoral competition (or a ‘yes-no’ referendum) for benefits of readers less acquainted with the material. We shall also indicate here how a large number of information sources can be aggregated in the form of a single information process, and how individual information source affects the overall information. We then examine the case of an election involving three candidates, and work out explicit formulae for the probabilities of six different outcomes (orderings of candidates) in a future election. Our focus here will be a first-past-the-post electoral system, although the fact that we have explicit formulae for all different scenarios means that the approach can be used in other electoral systems such as a proportional representation system. We then explore the impact of candidate’s positioning within the political spectrum. In this context it will be shown that among a polarized electorate the candidate with a centre-ground position can find that the probability of winning a future election is identically zero in a first-past-the-post electoral system, unless a significant amount of reliable information is revealed to create higher volatilities. We then discuss briefly how our model can be implemented in practice in an electoral competition. We conclude with a discussion on how closed-form formulae for the probabilities of the various different outcomes of an election can be obtained in the present scheme when there are more than three candidates.

## 2. Information-based modelling of electoral competition

Let us begin by examining the role of information in modelling an electoral competition. In a generic situation, voters will have a range of issues that concern them in deciding which candidate to choose. The policy positions of the candidates, if elected, on these issues, however, are known only partially to the voters. Nevertheless, as time progresses, more information about the candidates, or their views on different policy positions, are revealed, which in turn will shift voter perceptions. We can model this dynamics by use of the mathematical techniques of signal processing. Specifically, we model the position of candidate *l* on issue *k* by a random variable *X*^*k*,*l*^, taking a range of values labelling different policy positions on this issue, where the probability that *X*^*k*,*l*^ taking a given value represents the voter perception of the policy position of candidate *l* on this issue. Voters will then acquire partial information about the values of these random variables. For a fixed candidate and fixed issue, because the quantity of interest to voters is the candidate’s position on that issue, represented by the value of *X*^*k*,*l*^, we can think of this random variable as the ‘signal’ in a communication channel, which is superimposed with noise that represents, for instance, rumours, speculations, disinformation and so on. The arrival of information to voters can therefore be modelled in the form of a superposition of signal and noise. The idea we evoke here is that we regard the environment in which voters are living, in itself, as forming a communication channel, where noisy information is transmitted through newspapers, radio and television broadcasts, internet, word of mouth and so on. Then based on this partial information, voters will come up with best estimates for each of the factors. Candidates are then ranked in accordance with these estimates, reflecting the preferences of voters.

This concept has been formalized mathematically in [1] as a structural approach to modelling electoral competition, with an emphasis to determine the statistics of the impact of disinformation on democratic processes. For a better understanding of the information-based formalism it will be useful to explain the structural approach in more detail. The fundamental idea is to first model the information-providing process $\left\{\xi_{t}^{k,l}\right\}$ associated with the policy position *k* of candidate *l*. For example, if the noise that obscures the value of *X*^*k*,*l*^ is modelled by an additive Gaussian noise $\left\{B_{t}^{k,l}\right\}$, and if the rate at which information is revealed to the electorates at time *t* is given by $\sigma_{t}^{k,l}$, then the information process takes the form of a ‘signal-plus-noise’ decomposition,

2.1 $$\xi_{t}^{k,l}=X^{k,l}\int_{0}^{t}\sigma_{s}^{k,l}\;ds+B_{t}^{k,l}.$$

If we let $F_{t}$ denote the totality of information available to the electorates at time *t* generated by (2.1) for all *k*, *l*, then the best estimate people will arrive at about the *k*th policy position of candidate *l* is given by the conditional expectation ${\hat{X}}_{t}^{k,l}=E[X^{k,l}|F_{t}]$.

Next, for a given voter *m*, we let *w*_*m*,*k*_ denote the preference weight of that voter for issue *k*. Then we can identify the ‘score’ $S_{m}^{l}(t)$ assigned at time *t* by voter *m* for candidate *l*. For example, in a linear scoring system we have

2.2 $$S_{m}^{l}(t)=\sum_{k}w_{m,k}\;{\hat{X}}_{t}^{k,l}.$$

Then at time *T* of the election, voter *m* will choose the candidate with the highest score at that time. Because a large number of the voter preferences {*w*_*m*,*k*_} can be sampled from a distribution [1], in a structural approach it is possible to engage in a rather detailed issue-by-issue scenario analysis to identify optimal informational strategies, as well as making predictions of the statistics of a future election.

## 3. Reduced-form approach to electoral competition

An alternative ‘reduced-form’ approach has also been introduced in [1] and was further developed in [2]. The idea of a reduced-form approach is to aggregate a broad range of issues into a single random variable *X* that labels different candidates. We remark that the terminologies of structural versus reduced-form are derived from an analogous consideration in the modelling of credit risk in financial markets. Here, for a given cash flow associated with a given firm, one can attempt to go into a detailed structural analysis of that firm in identifying the risk associated with that cash flow. However, in most cases this is not feasible because relevant financial structures—for instance other cash flows linked to that firm—are far too complicated and often not even transparent. To remedy this issue, in credit risk modelling an alternative reduced-form approach has been introduced as a way for capturing the essence of credit risks without going into any of the structural details so that the method can be applied universally and systematically in practical applications.

In contrast to credit risk modelling, for an electoral competition a structural approach is entirely feasible. This is because the number of significant issues that are of concern to a large number of electorates is limited—perhaps a handful in this case, as opposed to thousands in the case of a typical credit product—and likewise the number of candidates is also limited. Nevertheless, the advantage of the reduced-form formalism, which will be explained in more detail now, is that just as in credit risk modelling, the mathematical analysis can be simplified considerably without losing many of the key features of the structural formalism. In this paper, we shall therefore develop the theory underlying a three-candidate race in the reduced-form approach.

In a reduced-form approach to modelling electoral competitions, a wide-ranging information relevant to deciding which candidate to choose is aggregated in the form of a single information process that represents abstractly the choice of the candidates. More specifically, in an election with *N* candidates we let *X* be the random variable taking the values {*x*_*k*_}, *k* = 1, 2, …, *N*, that label different candidates, with the probabilities {*p*_*k*_}. These probabilities represent the current opinion poll statistics. These statistics will change in time, in accordance with the revelation of information related to the candidates. Recall that voters wish to determine which candidate to vote for. Hence in a reduced-form model the random variable *X* plays the role of the ‘signal’.

As an elementary model let us assume that information concerning the candidates is revealed to the voters at a constant rate *σ*, and that wide-ranging noise is modelled by a Brownian motion {*B*_*t*_}. Then the flow of information takes the familiar signal-plus-noise form

3.1 $$\xi_{t}=\sigma Xt+B_{t}.$$

In a more realistic scenario, the information flow rate is time dependent, and in this case the signal component is modified to $X\int_{0}^{t}\sigma_{s}\;ds$. However, for simplicity we shall consider the special case in which *σ*_*t*_ = *σ* is constant, with the remark that all the results presented below can be extended to the time-dependent case without losing analytical tractability.

It is worth remarking that in a real election there are many information sources associated with the candidate choice *X*. We thus have the information process $\xi_{t}^{j}=\sigma_{j}Xt+B_{t}^{j}$ generated by the *j*th information source. However, without loss of generality we can aggregate different information sources in the form of a single information process (3.1). To see this, let us first consider a pair of information sources: $\xi_{t}^{1}=\sigma_{1}Xt+B_{t}^{1}$ and $\xi_{t}^{2}=\sigma_{2}Xt+B_{t}^{2}$. Letting $\rho =E[B_{t}^{1}B_{t}^{2}]$ denote the correlation of the two Brownian noise, it is an elementary fact that there exists a Brownian motion ${\bar{B}}_{t}$, independent of $B_{t}^{1}$, such that we have $B_{t}^{2}=\rho B_{t}^{1}+\sqrt{1-\rho^{2}}{\bar{B}}_{t}$. Defining

3.2 $$\bar{\sigma }=\frac{\sigma_{2}-\rho \sigma_{1}}{\sqrt{1-\rho^{2}}}\;\mathrm{and}\;{\bar{\xi }}_{t}=\bar{\sigma }Xt+{\bar{B}}_{t},$$

we deduce that the information generated jointly by $\xi_{t}^{1}$ and $\xi_{t}^{2}$ is equivalent to that generated jointly by $\xi_{t}^{1}$ and ${\bar{\xi }}_{t}$. Next, let us define

3.3 $$\sigma^{2}=\sigma_{1}^{2}+{\bar{\sigma }}^{2}\;\mathrm{and}\;B_{t}=\frac{1}{\sigma }(\sigma_{1}B_{t}^{1}+\bar{\sigma }{\bar{B}}_{t}),$$

and set

3.4 $$\xi_{t}=\sigma Xt+B_{t}\;\mathrm{and}\;\delta_{t}=\frac{\xi_{t}^{1}}{\sigma_{1}}-\frac{{\bar{\xi }}_{t}}{\bar{\sigma }}=\frac{B_{t}^{1}}{\sigma_{1}}-\frac{{\bar{B}}_{t}}{\bar{\sigma }}.$$

Then a short calculation shows that the information generated jointly by $\xi_{t}^{1}$ and $\xi_{t}^{2}$ is equivalent to that generated jointly by *ξ*_*t*_ and *δ*_*t*_, and that *ξ*_*t*_ and *δ*_*t*_ are independent. However, *δ*_*t*_ is independent of the random variable *X*. It follows that $E[X|\{\xi_{t}^{1},\xi_{t}^{2}\}]=E[X|\{\xi_{t}\}]$. In other words, the aggregate of the two information sources can be represented in the form of a single information process (3.1).

More generally, suppose that we have a series of information processes of the form

3.5 $$\begin{array}{cc} & \xi_{t}^{1}=\sigma_{1}Xt+B_{t}^{1}\\ & \;\;\;\;\vdots \\ & \xi_{t}^{n}=\sigma_{n}Xt+B_{t}^{n} \end{array}\}$$

available to the electorates about the choice of candidates, where the various noise processes ${\left\{B_{t}^{i}\right\}}_{i=1,\ldots ,n}$ in general may be mutually correlated, with the correlation matrix *ρ*_*ij*_. Then the aggregate of the *n* information processes (3.5) can be represented by means of a single information process of the form (3.1), where

3.6 $$\sigma^{2}=\frac{\sum_{i}^{n}\sigma_{i}^{2}\;\rho_{ii}^{-1}-2\sum_{i\neq j}\sigma_{i}\;\sigma_{j}\;\rho_{ij}^{-1}}{\det(\rho )}\;$$

represents the *effective information flow rate* and

3.7 $$B_{t}=\frac{1}{\sigma }\left(\sum_{i,j}^{n}\sigma_{i}\;\rho_{ij}^{-1}\;B_{t}^{i}\right)\;$$

represents the *effective noise*. Here $\rho_{ij}^{-1}$ denotes the *ij* element of the inverse correlation matrix. This observation shows that although the idea of representing a wide range of information flows in terms of a single information process at first may seem restrictive, in fact it is quite general.

Given our model (3.1) to represent the flow of information, the initial voter preference for a candidate, embodied in the *a priori* probability $p_{i}=P(X=x_{i})$, will change into the posterior preference $\pi_{it}=P(X=x_{i}|{\left\{\xi_{s}\right\}}_{0\leq s\leq t})$, which is just the conditional probability that candidate labelled by *x*_*i*_ should be chosen, given the information available up to time *t*. In the present example in which the information flow rate is constant, the information providing process {*ξ*_*t*_} is Markov, from which it follows that the conditional probability simplifies into $\pi_{it}=P(X=x_{i}|\xi_{t})$. Then by use of the Bayes formula

3.8 $$P(X=x_{i}|\xi_{t})=\frac{P(X=x_{i})\rho (\xi_{t}|X=x_{i})}{\sum_{\;j}P(X=x_{j})\rho (\xi_{t}|X=x_{j})},$$

along with the fact that the conditional density function *ρ*(*ξ*_*t*_|*X* = *x*_*i*_) for the random variable *ξ*_*t*_ is Gaussian and is given by

3.9 $$\rho (\xi |X=x_{i})=\frac{1}{\sqrt{2\pi t}}\exp\left(-\frac{{\left(\xi -\sigma x_{i}t\right)}^{2}}{2t}\right),$$

we deduce at once that

3.10 $$\pi_{it}=\frac{\;p_{i}\exp\left(\sigma x_{i}\xi_{t}-\frac{1}{2}\sigma^{2}x_{i}^{2}t\right)}{\sum_{j}p_{j}\exp\left(\sigma x_{j}\xi_{t}-\frac{1}{2}\sigma^{2}x_{j}^{2}t\right)}.$$

Incidentally, this expression in the context of signal processing is known as the Wonham filter [15] associated with the detection of a random drift of a Brownian motion; a problem that has also been explored more recently in different contexts [16,17].

## 4. Two-candidate scenario

Given the expression (3.10) for the *a posteriori* probability that the *i*th candidate should be chosen, we are able to ask a range of questions that link informational strategies to the election outcome. To this end, we determine first the probability that the *i*th candidate wins a future election, given (i) current support rate, and (ii) how information is managed from now to the election day, in the case of a two-candidate competition. In this case, we may let, without loss of generality, the random variable *X* labelling the two candidates be binary, taking the values 0 and 1. Let *p* be the probability that *X* = 0 and 1 − *p* be the probability that *X* = 1. Then the *a posteriori* probability that, say, candidate 0 being chosen when an arbitrary voter is sampled from the population is

4.1 $$\pi_{0t}=\frac{\;p}{p+(1-p)\exp\left(\sigma \xi_{t}-\frac{1}{2}\sigma^{2}t\right)}.$$

If the election were to take place in *T* years time from today (today always implies time *t* = 0), then the probability that candidate 0 should win the election, subject to current poll and how information is revealed from today to the election day, is therefore given by $P(\pi_{0T}>1/2)$.

It is important to note that the realized winning probability $P(\pi_{0T}>1/2)$ for candidate 0, as of today, can be very different from the current support rate *p*. To understand this, imagine that the election is to take place in a week, and that candidate 0 currently has 55% support. Then unless something radical happens—such as a revelation of a major scandal—it is likely that voter preferences will not change very much in one week, hence candidate 0 will receive nearly 55% of the votes to secure a victory. In other words, in such a scenario the realized probability of candidate 0 winning the future election is close to 100%, even though the support rate remains only 55%. Putting the matter differently, today’s poll statistics is not the predictor for the likelihood of winning a future election, although it can be used to calculate the likelihood.

One advantage of our approach is that we are able to derive an explicit formula for the probability of a given candidate winning the election that reflects this observation. The detailed derivation of the formula in the case of a two-candidate election is provided in [2], which we shall not reproduce here. Instead, we mention some key steps that will be exploited in the present context. Namely, that the denominator of the conditional probability (3.10) can be used to change probability measure P into a new measure Q such that under Q the information process {*ξ*_*t*_} is a standard Brownian motion [2]. Specifically, writing

4.2 $$\Phi_{t}=p+(1-p)\exp\left(\sigma \xi_{t}-\frac{1}{2}\sigma^{2}t\right)\;$$

for the measure-change martingale, we have

4.3 $$P\left(\pi_{0T}>\frac{1}{2}\right)=E^{P}\left[1\left\{\pi_{0T}>\frac{1}{2}\right\}\right]=E^{Q}\left[\Phi_{T}\;1\left\{\pi_{0T}>\frac{1}{2}\right\}\right].$$

Now the condition that *π*_0*T*_ > 1/2 is equivalent to the condition that

4.4 $$\frac{\xi_{T}}{\sqrt{T}}<\frac{\log\left(\frac{p}{1-p}\right)+\frac{1}{2}\sigma^{2}T}{\sigma \sqrt{T}},$$

but under Q the information process is a Brownian motion, and hence $\xi_{T}/\sqrt{T}$ is a standard normal random variable. It then follows at once that

4.5 $$P\left(\pi_{0T}>\frac{1}{2}\right)=p\;N(d^{+})+(1-p)\;N(d^{-}),$$

where

4.6 $$d^{\pm }=\frac{\log\left(\frac{p}{1-p}\right)\pm \frac{1}{2}\sigma^{2}T}{\sigma \sqrt{T}}$$

and

4.7 $$N(x)=\frac{1}{\sqrt{2\pi }}\int_{-\infty }^{x}\;e^{-\frac{1}{2}z^{2}}\;dz\;$$

denotes the cumulative normal distribution function. It is a curious coincidence that the winning probability of a candidate in a two-candidate election is essentially the same as the option pricing formula of Black and Scholes in financial modelling. The formula shows, for instance, that if candidate 0 has 55% support rate today and if the election is to take place in a week, then even if the information revelation rate is as large as, say, *σ* = 1.2, the winning probability will be about 89%; whereas if the information revelation rate is reduced to, say, *σ* = 0.5, then this probability increases to 99.8%. In other words, the model reflects the intuition described above. Putting it differently, formula (4.5) allows us to interpolate between today’s and future’s statistics.

**Figure 1.**
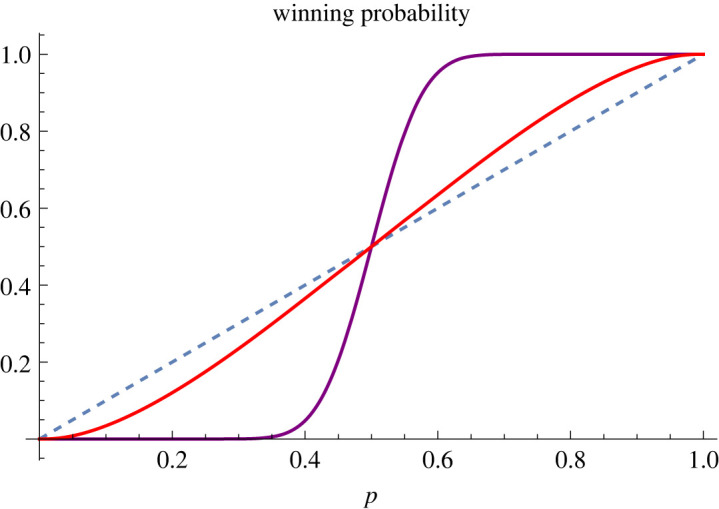
Winning likelihood. The probability that candidate 0 will win the election in 18 months (*T* = 1.5), as a function of the current support rate *p* for the candidate. The realized likelihood of winning a future election is always higher than today’s poll if *p* > 1/2; and conversely lower than the poll if *p* < 1/2. How much the winning probability deviates from the current poll depends on how much information is revealed over the next 18 months. Here, two examples are shown, corresponding to the values *σ* = 0.2 (in purple) and *σ* = 1.2 (in red).

We therefore see how an explicit formula (4.5) for a given candidate winning a future election can be obtained in the case of a two-candidate competition. The winning probability, more explicitly, depends on the following three ingredients: (i) the current support rate *p* for the candidate, (ii) the time *T* left to the election, and (iii) the rate *σ* at which information is revealed to the electorate from now until the election day. The only ‘control’ parameter at candidates’ disposal therefore is the information flow rate *σ*. To gain a better intuition here, therefore, let us examine how the winning probability depends on the current poll statistics for different values of *σ*. In figure 1 we plot the winning probability for candidate 0 as a function of the current support rate *p* for two different values of *σ*. It is evident that if very little information is revealed from today to the election day, then the current support rate will not change significantly so that the likelihood of winning the election is considerably higher (lower) than the current poll if it is higher (lower) than 50%. What this means is that in a two-candidate election, if a candidate is losing then it is in their interest to release as much information as possible to generate more volatility; whereas if the candidate is winning then it is in their interest to conceal as much information as possible. This situation may be empirically familiar to some election strategists. When there are three or more candidates, however, there are some non-trivial situations that can arise, as we shall discuss below.

## 5. Three-candidate electoral competition

In a reduced-form approach, when there are three candidates we let the random variable *X* take the values {*x*_*k*_} (*k* = 1, 2, 3), labelling the these candidates. Thus the event that *X* = *x*_3_, say, means that the third candidate is being selected as the ‘right’ choice, *if the voters were to have access to the information* {*ξ*_*t*_} *eternally*. That is, in this scenario, which occurs with probability *p*_3_, the sample path of *π*_3*t*_ will be such that we have *π*_3*t*_ → 1 as *t* → ∞. Of course, the election will take place earlier, so the vote share for the third candidate on the election day will instead be *π*_3*T*_, which may or may not be larger than the support rates of the other two candidates. Hence even in the event in which *X* = *x*_3_, in general this has little to do with the likelihood of the third candidate winning the election, unless the value of *σ* is unusually large.

As a matter of interpretation, to further clarify the meaning of the random variable *X*, we remark that our model set-up is such that the value of *X* will only be revealed based on the information process {*ξ*_*t*_} over an infinite time horizon, because

5.1 $$\lim_{t\to \infty }\frac{\xi_{t}}{\sigma t}=X$$

in a distributional sense. In other words, if hypothetically the electorates were to live forever, then they will all learn which of the candidates they should all be voting for. However, the election will take place considerably sooner at time *T*, at which point there has not been sufficient information generated, according to the information process (unless *σ* is very large). Thus the voter variability remains high, and the election outcome remains uncertain. It follows that our model is only of use until the election day, at which point it can be discarded, but this is all we need in order to interpolate the statistics between today and the election day.

Differently stated, in a typical election cycle the information-providing process {*ξ*_*t*_} ceases to exist after the election day (or, equivalently, the information flow rate parameter *σ* goes to zero, leaving behind nothing but noise), so the voters will never learn which candidate would have been the ‘right’ candidate. Hence in reality none of the *a posteriori* probabilities will actually converge to unity (except perhaps in certain extreme circumstances). This is because no one will have access to sufficient information to allow them to appropriately assign probabilities on counterfactual events: what would have happened if that person were elected rather than this person. Indeed, even in an event whereby a candidate who lost the election remains engaged towards a subsequent election, it is not always the case that much information about that candidate reaches the voters, as exemplified by an 18 August 1996 Newsweek article titled ‘Forgotten, but not gone’ about the then presidential candidate Ross Perot [18].

In the case of a three-candidate race, there are six possible ordering scenarios for the vote share. Hence in order to determine the probability that the third candidate, say, wins the election, we need to work out the probabilities for individual ordering. This follows because the current probability for the third candidate to win the election is given by $P(\pi_{3T}>\pi_{2T}>\pi_{1T})+P(\pi_{3T}>\pi_{1T}>\pi_{2T})$; and similarly for the other candidates to win the election. To this end, we note from (3.10) that for any *j* ≠ *k* the event *π*_*kT*_ > *π*_*jT*_ holds true if and only if

5.2 $$p_{k}\exp\left(\sigma x_{k}\;\xi_{T}-\frac{1}{2}\sigma^{2}x_{k}^{2}T\right)>p_{j}\exp\left(\sigma x_{j}\;\xi_{T}-\frac{1}{2}\sigma^{2}x_{j}^{2}T\right).$$

This condition can easily be solved for a condition on *ξ*_*T*_, provided that the ordering of the numbers {*x*_*i*_} are given. Without loss of generality we may assume that *x*_3_ > *x*_2_ > *x*_1_. Then from (5.2) it follows that *π*_*kT*_ > *π*_*jT*_ holds if and only if

5.3 $$\xi_{T}>\frac{\log(p_{j}/p_{k})+\frac{1}{2}\sigma^{2}(x_{k}^{2}-x_{j}^{2})T}{\sigma (x_{k}-x_{j})},$$

provided that *x*_*k*_ > *x*_*j*_. Otherwise, the inequality is reversed.

To proceed, let us introduce the notation

5.4 $$z_{kj}=\frac{\log(p_{j}/p_{k})+\frac{1}{2}\sigma^{2}(x_{k}^{2}-x_{j}^{2})T}{\sigma (x_{k}-x_{j})}.$$

Then a short calculation shows, on account of the symmetry property *z*_*kj*_ = *z*_*jk*_, that

$$\begin{array}{rl} & \pi_{3T}<\pi_{2T}<\pi_{1T}\Leftrightarrow \xi_{T}<\min\{z_{12},z_{23}\},\\ & \pi_{2T}<\pi_{3T}<\pi_{1T}\Leftrightarrow z_{23}<\xi_{T}<z_{31}\;(\text{if}\;z_{23}<z_{31}),\\ & \pi_{3T}<\pi_{1T}<\pi_{2T}\Leftrightarrow z_{12}<\xi_{T}<z_{31}\;(\text{if}\;z_{12}<z_{31}),\\ & \pi_{1T}<\pi_{3T}<\pi_{2T}\Leftrightarrow z_{31}<\xi_{T}<z_{23}\;(\text{if}\;z_{31}<z_{23}),\\ & \pi_{2T}<\pi_{1T}<\pi_{3T}\Leftrightarrow z_{31}<\xi_{T}<z_{12}\;(\text{if}\;z_{31}<z_{12}),\\ \mathrm{and}\;\;\;\;\; & \pi_{1T}<\pi_{2T}<\pi_{3T}\Leftrightarrow \max\{z_{12},z_{23}\}<\xi_{T}. \end{array}$$

Note here that, for example, the event *π*_2*T*_ < *π*_3*T*_ < *π*_1*T*_ cannot be realized if *z*_23_ > *z*_31_, and similarly for other three intermediate cases. With these conditions at hand, let us note that the probability $P(a<\xi_{T}<b)$ for any *a* < *b* can be worked out by changing the probability measure. Specifically, we use the common denominator

5.5 $$\Phi_{t}=\sum_{\;j=1}^{3}p_{j}\exp\left(\sigma x_{j}\xi_{t}-\frac{1}{2}\sigma^{2}x_{j}^{2}t\right)$$

to effect a measure change P→Q so that under Q the information process {*ξ*_*t*_} is a standard Brownian motion. Then we have

5.6 $$\begin{array}{rl} P(a<\xi_{T}<b) & =E^{Q}[\Phi_{T}1\{a<\xi_{T}<b\}]\\ & =E^{Q}\left[\sum_{\;j=1}^{3}p_{\;j}\exp\left(\sigma x_{\;j}\xi_{T}-\frac{1}{2}\sigma^{2}x_{\;j}^{2}T\right)1\{a<\xi_{T}<b\}\right]\\ & =\sum_{\;j=1}^{3}p_{\;j}\left[N\left(\frac{b-\sigma x_{\;j}T}{\sqrt{T}}\right)-N\left(\frac{a-\sigma x_{\;j}T}{\sqrt{T}}\right)\right]. \end{array}$$

With these results at hand, we are able to work out the probabilities for the six possible outcomes. For concreteness, let us write them down explicitly here. They are

5.7 $$P(\pi_{3T}<\pi_{2T}<\pi_{1T})=\sum_{\;j=1}^{3}p_{\;j}\left[N\left(\frac{z_{12}-\sigma x_{\;j}T}{\sqrt{T}}\right)1\{z_{12}<z_{23}\}+N\left(\frac{z_{23}-\sigma x_{\;j}T}{\sqrt{T}}\right)1\{z_{12}\geq z_{23}\}\right]$$

and

5.8 $$P(\pi_{2T}<\pi_{3T}<\pi_{1T})=\sum_{\;j=1}^{3}p_{\;j}\left[N\left(\frac{z_{31}-\sigma x_{\;j}T}{\sqrt{T}}\right)-N\left(\frac{z_{23}-\sigma x_{\;j}T}{\sqrt{T}}\right)\right]1\{z_{23}<z_{31}\},$$

together determine the probability of the first candidate winning,

5.9 $$P(\pi_{3T}<\pi_{1T}<\pi_{2T})=\sum_{\;j=1}^{3}p_{\;j}\left[N\left(\frac{z_{31}-\sigma x_{\;j}T}{\sqrt{T}}\right)-N\left(\frac{z_{12}-\sigma x_{\;j}T}{\sqrt{T}}\right)\right]1\{z_{12}<z_{31}\}$$

and

5.10 $$P(\pi_{1T}<\pi_{3T}<\pi_{2T})=\sum_{\;j=1}^{3}p_{\;j}\left[N\left(\frac{z_{23}-\sigma x_{\;j}T}{\sqrt{T}}\right)-N\left(\frac{z_{31}-\sigma x_{\;j}T}{\sqrt{T}}\right)\right]1\{z_{31}<z_{23}\},$$

together determine the probability of the second candidate winning, and

5.11 $$P(\pi_{2T}<\pi_{1T}<\pi_{3T})=\sum_{\;j=1}^{3}p_{\;j}\left[N\left(\frac{z_{12}-\sigma x_{\;j}T}{\sqrt{T}}\right)-N\left(\frac{z_{31}-\sigma x_{\;j}T}{\sqrt{T}}\right)\right]1\{z_{31}<z_{12}\}$$

and

5.12 $$\begin{array}{rl} & P(\pi_{1T}<\pi_{2T}<\pi_{3T})\\ & \;=\sum_{\;j=1}^{3}p_{i}\left[N\left(-\frac{z_{23}-\sigma x_{\;j}T}{\sqrt{T}}\right)1\{z_{12}<z_{23}\}+N\left(-\frac{z_{12}-\sigma x_{\;j}T}{\sqrt{T}}\right)1\{z_{12}\geq z_{23}\}\right], \end{array}$$

together determine the probability of the third candidate winning. In this way, we obtain an explicit formula for each of the candidate winning the future election, as functions of (i) the current support rates {*p*_*j*_} for the candidates, (ii) the time *T* left to the election, (iii) the rate *σ* at which information is revealed to the electorate, and (iv) the choice of the candidate labels {*x*_*j*_}.

**Figure 2.**
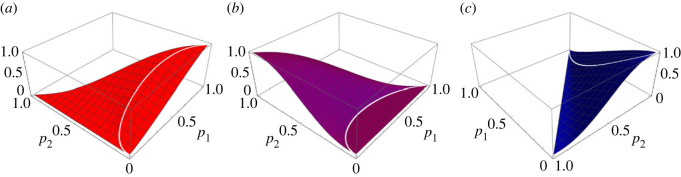
Winning probabilities as functions of (*p*_1_, *p*_2_). The probabilities of winning a future election to take place in 1 year's time (*T* = 1), when the information flow rate is set at *σ* = 1, are plotted here for the parameter choice (*x*_1_, *x*_2_, *x*_3_) = (1, 2, 3). The forms of the probabilities for candidate 1 (*a*, in red) and candidate 3 (*c*, in blue) are entirely symmetric. However, the behaviour of the probability for candidate 2 (*b*, in purple) is slightly different in that there is a region in the parameter space (*p*_1_, *p*_2_) of the current support rates for which the probability of candidate 2 winning is identically zero. We will have more to say about this in the next section.

In figure 2 we sketch the behaviours of the winning probabilities for the three candidates as functions of the current support rates (*p*_1_, *p*_2_) for the first two candidates. One distinguishing feature here, as compared with the results for two-candidate scenario, is the dependence on the information flow rate *σ*. In the two-candidate case, for a given level (*p*, 1 − *p*) of current support, the winning probabilities are either increasing or decreasing in *σ*. That is, if the candidate is leading the poll, then it is best not to reveal information, and conversely for the other candidate. In the three-candidate case, depending on the level (*p*_1_, *p*_2_, 1 − *p*_1_ − *p*_2_) of current support, the winning probabilities can lack monotonicity. That is, there are values of (*p*_1_, *p*_2_, 1 − *p*_1_ − *p*_2_) for which a candidate will benefit from, say, increasing the information flow rate slightly to enhance the probability of winning the future election, but if it is increased too much, then this will result in decreasing the probability again. It follows that in a three-candidate race, the optimal strategy of controlling information can be quite non-trivial for certain values of the current support rates (*p*_1_, *p*_2_, 1 − *p*_1_ − *p*_2_).

**Figure 3.**
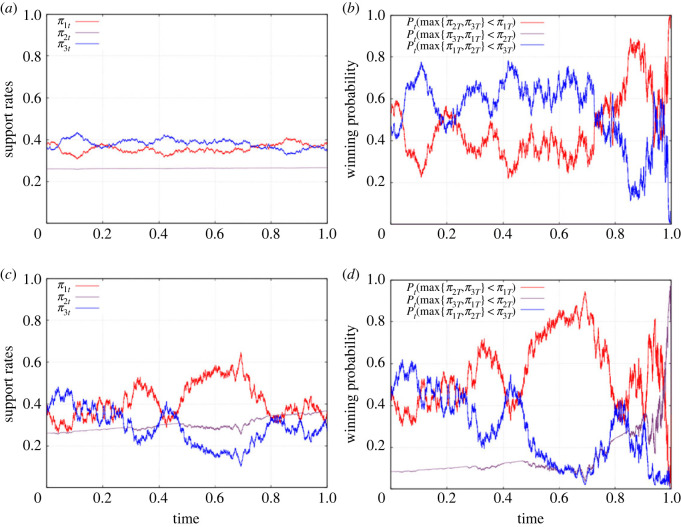
Dynamical behaviours of the poll statistics {*π*_*it*_} and the corresponding winning probabilities. Sample paths for the support rates (*π*_1*t*_, *π*_2*t*_, *π*_3*t*_) for the three candidates are shown in (*a*,*c*). The corresponding winning probability processes for each candidate are shown in (*b*,*d*). The parameters are chosen as (*x*_1_, *x*_2_, *x*_3_) = (1, 2, 3) for the values of the random variable *X*, (*p*_1_, *p*_2_, *p*_3_) = (0.38, 0.26, 0.36) for the current support level so that the electorates are slightly polarized, and *T* = 1 year for the time left to the election day. Panels (*a*,*b*) correspond to the value *σ* = 0.25 for the information flow rate. In this case, the probability for the second candidate to win the election is identically zero. For a comparison, the corresponding results for the choice *σ* = 1 are plotted in (*c*,*d*), in which the second candidate narrowly secures a victory.

Although we have worked out here the initial (time *t* = 0) probability of a given candidate winning the election, it is straightforward to work out the corresponding conditional probabilities, such as $P(\pi_{1T}<\pi_{2T}<\pi_{3T}|\xi_{t})$ and so on, that depend on how information has been unravelled up to time *t*. Then we are able to simulate not just the support rates {*π*_*it*_} but also the realized winning probabilities, as illustrated in figure 3.

## 6. Representing political spectrum

It is important to emphasize the fact that while in a two-candidate electoral competition the choice of the labelling numbers {*x*_0_, *x*_1_} can be made arbitrarily, this is no longer the case if there are more than two candidates. The reason can be explained as follows.

We note first that from a signal detection perspective, whether the signal is an unknown random variable *X* or a (known) constant addition of an unknown *X*, there is no difference in the inference. This follows from the fact that the filtration generated by *σX t* + *B*_*t*_ is identical to that generated by *σ*(*X* + *c*)*t* + *B*_*t*_, where *c* is a known constant. Hence the only quantities of relevance in the choice of *X* are the gaps *ω*_*ij*_ = *x*_*j*_ − *x*_*i*_. Now in the binary case there is only one such gap *ω* = *x*_1_ − *x*_0_, but scaling the gap according to *ω* → *λω* is entirely equivalent to scaling the information flow rate *σ* → *λσ*. Putting it differently, the scaling *ω* → *λω* can be compensated by the scaling *σ* → *λ*^−1^*σ* so that *ω* can be chosen arbitrarily by regarding *σ* as the variable parameter.

In the case of an election with three candidates, there are three gaps, *ω*_12_, *ω*_23_ and *ω*_31_, with one constraint *ω*_12_ + *ω*_23_ + *ω*_31_ = 0. Hence there are two independent scaling parameters, which cannot be simultaneously absorbed by scaling *σ*. It follows that the probability of a given candidate winning the election is dependent on the choice of the gaps {*ω*_*ij*_}. Alternatively stated, there is a natural ordering (i.e. spectrum) encoded in the random variable *X* representing the candidates. In particular, the three candidates cannot be placed on an equal footing, for, while it is possible to set *ω*_12_ = *ω*_23_, it is not possible to set *ω*_12_ = *ω*_23_ = *ω*_31_.

We can take advantage of this feature of the model by observing that there is a long-established notion of a ‘political spectrum’ in an electoral process, and we can encode this information naturally in the choices of the gaps {*ω*_*ij*_}. Thus, for example, if candidate 1 is on the left, candidate 2 is moderately on the right, and candidate 3 is further on the right, then we can let *ω*_12_ > *ω*_23_ to capture this composition; and similarly for other situations. Realizing this, we see that the choice of the gaps {*ω*_*ij*_} is not up to the modeller, but it is up to the candidates in terms of where they place themselves in the political spectrum.

With this in mind, we find that there are circumstances in which taking the political centre ground leads to a disadvantage. This follows from the observation that the probability for candidate 2 to win the election is identically zero if *z*_12_ > *z*_31_ > *z*_23_, while such a constraint does not exist for the candidate to the left or to the right. Note, however, that this does not mean that taking the centre ground is always disadvantageous—it merely shows that in certain situations, having popular competitors to both the left and the right can be fatal. In particular, such a trap for candidate 2 can be created among a politically polarized set of voters so that *p*_2_ < *p*_1_, *p*_3_ holds while at the same time *p*_1_ ∼ *p*_3_.

More specifically, a calculation shows that the condition *z*_12_ > *z*_31_ > *z*_23_ can be translated into a bound on the information flow rate *σ* as follows:

6.1 $$\sigma^{2}<\frac{2}{\omega_{12}\omega_{23}\omega_{31}T}\;\min\left\{\omega_{12}\log\frac{\;p_{1}}{p_{3}}-\omega_{13}\log\frac{\;p_{1}}{p_{2}},\;\omega_{32}\log\frac{\;p_{1}}{p_{3}}-\omega_{13}\log\frac{\;p_{3}}{p_{2}}\right\}.$$

**Figure 4.**
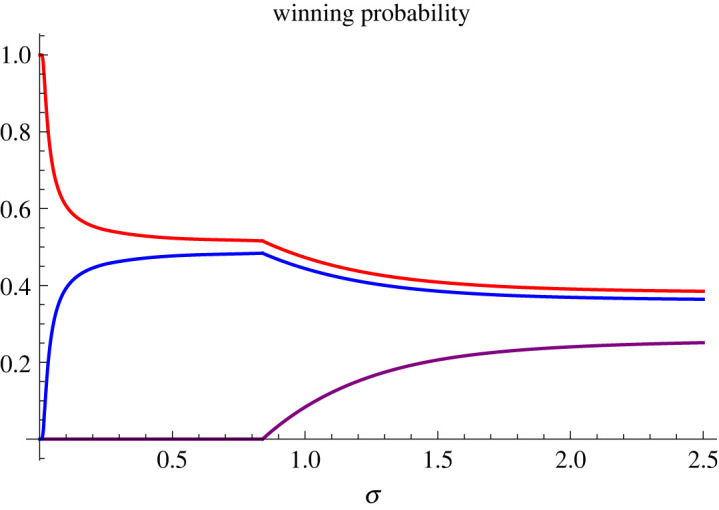
Winning probabilities as functions of *σ*. The probability of winning an election in 1 year's time (*T* = 1), as a function of the information flow rate *σ*, is shown for the three candidates, labelled according to *x*_1_ = 1, *x*_2_ = 2 and *x*_3_ = 3. The current poll statistics are taken to be *p*_1_ = 0.38 for the first candidate on the left (red), *p*_2_ = 0.26 for the second candidate taking the centre ground (purple), and *p*_3_ = 0.36 for the third candidate on the right (blue).

That is, provided that the inequality (6.1) holds, the probability of candidate 2 winning the election is identically zero. This situation is illustrated in figure 4. A closer inspection shows that if *p*_2_ is small, then the bound on *σ* can be large. It follows that among a politically polarized electorate, the only way in which a candidate holding the centre ground has any chance of winning the election is to ensure that a lot of reliable information is revealed so as to increase the volatility of the poll statistics {*π*_*it*_}.

**Figure 5.**
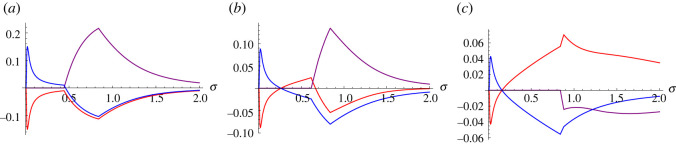
Gains in winning probabilities as functions of *σ*. If the political positioning (*x*_1_, *x*_2_, *x*_3_) = (1, 2, 3) considered in figure 4 is shifted, how would that affect the winning probabilities? Here, the difference of the resulting winning probabilities to the one in figure 4 is shown for three different cases: (*x*_1_, *x*_2_, *x*_3_) = (0.1, 2, 3.9) (*a*), (*x*_1_, *x*_2_, *x*_3_) = (1, 2, 3.9) (*b*) and (*x*_1_, *x*_2_, *x*_3_) = (1.5, 2, 3.9) (*c*). Other parameters are kept unchanged (*p*_1_ = 0.38, *p*_2_ = 0.26, *p*_3_ = 0.36, and *T* = 1). If the difference is negative, then clearly the shift is disadvantageous. The result shows that among a polarized electorate, if the candidate on the left of the political spectrum leans further to the left and the candidate on the right leans further to the right, then this is generally disadvantageous for both. However, if the competition is dominated by noise (small *σ* values), then the candidate on the right can benefit by leaning further to the right.

We now ask a related question on positioning within a political spectrum. For this purpose, we shall take the convention that if *x*_*j*_ < *x*_*k*_ then candidate *j* is placed politically to the left of candidate *k*. The question that we ask here more specifically is whether the winning probability can be enhanced by leaning further to the left or to the right. The answer will be dependent on the various parameter values, but let us consider the politically polarized case as shown in figure 4. In this case, if we keep the value of *x*_2_ unchanged but increase *x*_3_ and simultaneously decrease *x*_1_, then we find that the probability of winning the election for the candidate on the left decreases for all values of *σ*. However, for the candidate on the right, the situation is a little more complex. When the election process is overshadowed by noise (i.e. small *σ* values), the winning probability of the candidate on the right can be enhanced considerably by leaning further to the right; whereas if the election process is not dominated by noise, then the winning probability decreases by leaning further to the right. Hence in this scenario, there is no advantage for the candidate on the left to lean further to the left, but the candidate on the right has the advantage of turning more extreme, provided that the noise level is sufficiently high. If, however, the candidate misjudges the level of noise, then this strategy will backfire. Some examples illustrating this feature are given in figure 5, which shows, for example, that if the candidate on the right leans further to the right, while the candidate on the left leans slightly to the right, then there is a significant benefit to the candidate on the left, provided that the level of noise is not overwhelming.

## 7. Calibration and implementation

As regards the practical implementation of the model, let us examine the model parameters that can be calibrated, and those that can be controlled. To this end, the current support rates {*p*_*j*_} can be fixed from today’s poll, whereas *T* is fixed by the date of the election. The variables {*x*_*j*_} that the random variable *X* can take can then be fixed, up to an overall scale that can be absorbed into *σ*, by the relative positioning of the candidates within the political spectrum. A candidate, in particular, will have the choice for their own value of the number *x*_*k*_, but will not have control over the positioning of other candidates.

Finally, as for the information flow rate *σ*, its value cannot be controlled by any individual, but its current value can be estimated by studying the time series for the poll statistics. This follows from the fact that the volatility of the support rates {*π*_*it*_} is given by *σ*. Hence a historic estimate can be used to fix the value of *σ*. Alternatively, from the odds of election betting it is possible to work out the implied volatility, which can be used to fix the value of *σ*.

Having fixed all the parameters, the model can be used to interpolate the statistics from today to the election day. If the resulting probability of a given candidate winning the election appears undesirable, then that candidate will have small room to manoeuvre so as to increase the success probability. First, the candidate can adjust their positioning within the political spectrum. Of course other candidates may also adjust their positions as a consequence of this, but everything else being the same, our formula shows in which way the candidate should position themselves within the political spectrum.

The other variable that a candidate can adjust is the information flow rate. While no individual can fix the value of *σ*, the result of (3.6) shows in which way an individual flow rate *σ*_*k*_ affects the overall value of *σ*. In particular, *σ* is a monotonic function of each *σ*_*k*_, so increasing the value of any one of *σ*_*k*_ will increase the overall value of *σ*, and similarly decreasing the value of *σ*_*k*_ will decrease *σ*. Again, other candidates, or other information source such as the press, may adjust their information revelation rate as a result to counterbalance the impact. This, however, is just a fact about a democratic process—no one candidate can control its outcome. Nevertheless, our framework offers an immediately implementable procedure for guiding the candidates to identify which informational strategy will increase their chances of success, if everything else remained the same.

## 8. Discussion

We have examined in some detail the probability of a candidate winning a future election, and how it is affected by control variables such as the level of information revelation, or noise, and the positioning of the candidates within the political spectrum, in the case of an electoral competition involving three candidates. It should be evident that a closed form expression for a given candidate winning a future election can be obtained when there are more than three candidates. For example, if there are four candidates, then there are 24 different ways in which the support rates for the candidates on the election day can be ordered, e.g. *π*_2*T*_ < *π*_3*T*_ < *π*_1*T*_ < *π*_4*T*_ and so on. Each one of these will give rise to a bound on the random variable *ξ*_*T*_ in the form of $\xi_{T}\in D_{2314}$ for some domain $D_{2314}$ on the real line. (The analogue of these domains in the case of a three-candidate electoral competition would be $D_{231}=[z_{23},z_{31}]$, and so on.) The probability of this event being realized is therefore given by

8.1 $$P(\pi_{2T}<\pi_{3T}<\pi_{1T}<\pi_{4T})=E^{Q}\left[\sum_{\;j=1}^{4}p_{\;j}\exp\left(\sigma x_{\;j}\xi_{T}-\frac{1}{2}\sigma^{2}x_{\;j}^{2}T\right)1\{\xi_{T}\in D_{2314}\}\right],$$

but because *ξ*_*T*_ under Q is Gaussian with mean zero and variance *T*, this expectation can easily be worked out. By repeating the procedure for the five other domains $D_{2134}$, $D_{3124}$, $D_{3214}$, $D_{1234}$ and $D_{1324}$, and adding the results, we obtain the probability of the fourth candidate winning the election. Evidently, an analogous calculation can be performed for each of the other candidates to work out their success probabilities.

One of the non-trivial features that emerges when the number of candidates is greater than two is that there is a disadvantage for candidates positioning in the middle of the political spectrum, in a situation where the voters do not have strong preferences on centre grounds. An analogous property is seen also in the structural approach. The mathematical reason underlying this feature is as follows. If we label the *N* candidates such that *x*_1_ < *x*_2_ < · · · < *x*_*N*_, then for each fixed *T* and *σ* we find that *π*_1*T*_(*ξ*_*T*_), viewed as a function of *ξ*_*T*_, is monotonically decreasing in *ξ*_*T*_ without bound in the range [0, 1] and *π*_*NT*_(*ξ*_*T*_) is monotonically increasing in *ξ*_*T*_ without bound in the range [0, 1]. However, for any *k* ≠ 1, *N*, the function *π*_*kT*_(*ξ*_*T*_), which gives the support rate for the *k*th candidate on the election day, is unimodal and has a maximum value at $\xi_{T}=\xi_{k}^{*}$ that is strictly less than one, where $\xi_{k}^{*}$ is the unique solution to the equation

8.2 $$\sum_{\;j=1}^{N}(x_{j}-x_{k})p_{j}\exp\left(\sigma x_{j}\xi_{k}^{*}-\frac{1}{2}\sigma^{2}x_{j}^{2}T\right)=0.$$

**Figure 6.**
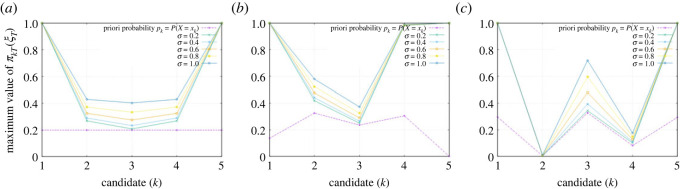
Maximum attainable support rates $\pi_{kT}(\xi_{k}^{*})$ for the five candidates. For a range of values for the information flow rate *σ*, the maximum values of {*π*_*kT*_} on the election day are shown by the dots, interpolated by lines to make the comparison easy. The current support rates {*p*_*k*_} are given by the bottom values (in purple). In (*a*), the five candidates are all assumed to have an equal support rate of 20%, whereas they are chosen at random in (*b*,*c*). The results show how the maximum attainable support rates for different candidates vary rather dramatically, depending on the existence of candidates having different political leanings and their associated current support rates. The parameters are chosen to be (*x*_1_, *x*_2_, *x*_3_, *x*_4_, *x*_5_) = (1, 2, 3, 4, 5) for the positioning of the candidates and *T* = 1 year for time left to the election.

If at least one of the variables *p*_*k*_, *σ*, or *T* is large, then the upper bound on *π*_*kT*_ will be close to one, so there is little concern for the candidate, but otherwise, the upper bound can be smaller than 1/*N*. In the latter case, whatever information is to be circulated, the probability of the *k*th candidate winning a future election is identically zero. When there are many candidates, the threshold value 1/*N* is small, but if there are only three or four candidates, then this effect is highly non-trivial and should not be ignored. Indeed, as we have seen in the case of a three-candidate race in figure 4, there is a wide range of values for the information flow rate *σ* for which there is no chance for the second candidate to win the election. In figure 6 we show the maximum attainable support rates for each candidate in the case of an election with five candidates. Figure 6*b* shows that while the initial support rates *p*_2_ and *p*_4_ for the centre left and centre right candidates are very close, the existence of a far-right candidate with negligible current support level implies that the maximum attainable value of *π*_4*T*_ is close to 1, whereas the existence of a far-left candidate with a moderate current support level implies that the maximum attainable value of *π*_2*T*_ is considerably lower than that of *π*_4*T*_.

Another non-trivial feature that emerges when there are more than two candidates is the subtle dependence of the winning probability on the positioning of the candidates within the political spectrum. We have merely uncovered for illustration a small number of features shown in figures 5 and 6, but a detailed sensitivity study of the winning probabilities on the spectrum {*x*_*j*_} is entirely feasible on account of the fact that we have closed-form expressions for these probabilities.

We conclude by remarking how our model might be extended. Throughout the paper we have assumed that the information revelation rate *σ* is constant, but in reality this is never the case. The analysis presented here nevertheless extends naturally to the case in which {*σ*_*t*_} has a deterministic time dependency. Specifically, in this case the candidate support rates take the form

8.3 $$\pi_{it}=\frac{\;p_{i}\exp\left(x_{i}\int_{0}^{t}\sigma_{s}\;d\xi_{s}-\frac{1}{2}x_{i}^{2}\int_{0}^{t}\sigma_{s}^{2}\;ds\right)}{\sum_{j}p_{j}\exp\left(x_{j}\int_{0}^{t}\sigma_{s}\;d\xi_{s}-\frac{1}{2}x_{j}^{2}\int_{0}^{t}\sigma_{s}^{2}\;ds\right)}.$$

Then the relevant random variable *ξ*_*T*_ is replaced with $\int_{0}^{T}\sigma_{s}\;d\xi_{s}$, which remains Gaussian under Q, so the various probabilities can still be worked out while taking into account a time-varying informational strategy {*σ*_*t*_}. In this way, impacts of a range of time-dependent informational strategies can be investigated. Of course, the information process {*ξ*_*t*_} is meant to represent the aggregate of the various information sources, and so is the variable *σ* (see [19] for how the aggregated information flow rate is related to that of the individual information source); whether it is time dependent or not. Thus, no one candidate has the access to control the overall value of *σ*. Nevertheless, the idea is that each candidate can influence the value of *σ*, which in turn will modify the likelihoods of the candidates winning the election.

## Data Availability

For generating figures, codes were created to either plot known functions or to simulate known stochastic processes derived in the paper. All codes are attached as electronic supplementary material [20]. Figures 1, 2, 4 and 5 were created using Mathematica code, included as a single file, while figures 3 and 6 were created using C, and are included as two separate files.

## References

[1] Brody DC, Meier DM. 2022 Mathematical models for fake news. In *Financial informatics: an information-based approach to asset pricing* (eds DC Brody *et al.*). Singapore: World Scientific. (First appeared in 2018 in https://arxiv.org/abs/1809.00964).

[2] Brody DC. 2019 Modelling election dynamics and the impact of disinformation. Inf. Geometry **2**, 209-230. (10.1007/s41884-019-00021-2)

[3] Brody DC. 2022 Noise, fake news, and tenacious Bayesians. Front. Psychol. **13**, 797904. (10.3389/fpsyg.2022.797904) 35602675 PMC9115576

[4] Davis OA, Hinich MJ, Ordeshook PC. 1970 An expository development of a mathematical model of the electoral process. Am. Pol. Sci. Rev. **64**, 426-448. (10.2307/1953842)

[5] Shepsle KA. 1972 The strategy of ambiguity: uncertainty and electoral competition. Am. Pol. Sci. Rev. **66**, 555-568. (10.2307/1957799)

[6] Harrington Jr JE. 1982 Modelling the role of information in elections. Math. Comput. Modell. **16**, 133-145.

[7] McKelvey RD, Ordeshook PC. 1985 Elections with limited information: a fulfilled expectations model using contemporaneous poll and endorsement data as information sources. J. Econ. Theory **36**, 55-85. (10.1016/0022-0531(85)90079-1)

[8] Coughlin PJ. 1992 Probabilistic voting theory. Cambridge, UK: Cambridge University Press.

[9] Feddersen T, Pesendorfer W. 1997 Voting behaviour and information aggregation in elections with private information. Econometrica **65**, 1029-1058. (10.2307/2171878)

[10] Fowler A, Margolis M. 2014 The political consequences of uninformed voters. Electoral Stud. **34**, 100-110. (10.1016/j.electstud.2013.09.009)

[11] Rowden J, Lloyd DJB, Gilbert N. 2014 A model of political voting behaviours across different countries. Physica A **413**, 609-625. (10.1016/j.physa.2014.07.022)

[12] Coughlin PJ. 2015 Probabilistic voting in models of electoral competition. In *Handbook of social choice and voting* (eds JC Heckelman, NR Miller). Cheltenham, UK: Edward Elgar Publishing Ltd.

[13] Yang VC, Abrams DM, Kernell G, Motter AE. 2020 Why are U.S. parties so polarized? A ‘satisficing’ dynamical model. SIAM Rev. **62**, 646-657. (10.1137/19M1254246)

[14] Brody DC, Hughston LP, Macrina A (eds). 2022 Financial informatics: an information-based approach to asset pricing. Singapore: World Scientific.

[15] Wonham WM. 1965 Some applications of stochastic differential equations to optimal nonlinear filtering. J. Soc. Ind. Appl. Math. A **2**, 347-369. (10.1137/0302028)

[16] Buonaguidi B. 2023 An optimal sequential procedure for determining the drift of a Brownian motion among three values. Stoch. Process. Appl. **129**, 320-349. (10.1016/j.spa.2023.02.001)

[17] Ekström E, Vaicenavicius J. 2015 Bayesian sequential testing of the drift of a Brownian motion. ESAIM Probab. Stat. **19**, 626-648. (10.1051/ps/2015012)

[18] Newsweek staff. 1996 Forgotten, but not gone. *Newsweek*. 18 August. See https://www.newsweek.com/forgotten-not-gone-177454.

[19] Brody DC, Law YT. 2015 Pricing of defaultable bonds with random information flow. Appl. Math. Finance **22**, 399-420. (10.1080/1350486X.2015.1050151)

[20] Brody DC, Yuasa T. 2023 Three-candidate election strategy. Figshare. (10.6084/m9.figshare.c.6837517) PMC10523069 37771969

